# Drought-induced dieback of *Pinus nigra*: a tale of hydraulic failure and carbon starvation

**DOI:** 10.1093/conphys/coz012

**Published:** 2019-05-15

**Authors:** Tadeja Savi, Valentino Casolo, Anna Dal Borgo, Sabine Rosner, Valentina Torboli, Barbara Stenni, Paolo Bertoncin, Stefano Martellos, Alberto Pallavicini, Andrea Nardini

**Affiliations:** 1 Dipartimento di Scienze della Vita, Università di Trieste, Via L. Giorgieri 10, Trieste, Italia; 2 Division of Viticulture and Pomology, Department of Crop Sciences, University of Natural Resources and Life Sciences, Konrad Lorenz Strasse 24, Tulln, Austria; 3 Dipartimento di Scienze Agro-Alimentari, Ambientali e Animali, Università di Udine, Via delle Scienze 91, Udine, Italia; 4 Institute of Botany, University of Natural Resources and Life Sciences, Gregor Mendel Strasse 33, Vienna, Austria; 5 Dipartimento di Scienze Ambientali, Informatica e Statistica, Università Ca’ Foscari Venezia, Via Torino 155, Venezia Mestre, Italia

**Keywords:** Black pine, carbon metabolism, drought, plastome, rooting depth, water status

## Abstract

Ongoing climate change is apparently increasing tree mortality rates, and understanding mechanisms of drought-induced tree decline can improve mortality projections. Differential drought impact on conspecific individuals within a population has been reported, but no clear mechanistic explanation for this pattern has emerged. Following a severe drought (summer 2012), we monitored over a 3-year period healthy (H) and declining (D) *Pinus nigra* trees co-occurring in a karstic woodland to highlight eventual individual-specific physiological differences underlying differential canopy dieback. We investigated differences in water and carbon metabolism, and xylem anatomy as a function of crown health status, as well as eventual genotypic basis of contrasting drought responses. H and D trees exploited the same water pools and relied on similar hydraulic strategies to cope with drought stress. Genetic analyses did not highlight differences between groups in terms of geographical provenance. Hydraulic and anatomical analyses showed conflicting results. The hydraulic tracheid diameter and theoretical hydraulic conductivity were similar, but D trees were characterized by lower water transport efficiency, greater vulnerability to xylem conduit implosion and reduced carbohydrate stores. Our results suggest that extreme drought events can have different impacts on conspecific individuals, with differential vulnerability to xylem embolism likely playing a major role in setting the fate of trees under climate change.

## Teaser

Extreme summer droughts induced differential impacts in a population of Pinus nigra trees. We analyzed eventual physiological differences between desiccated and healthy trees. Desiccated trees were more vulnerable to drought-induced xylem embolism, and were also suffering from depletion of carbohydrate reserves. Our data suggest that genotypic or phenotypic intraspecific variability can play fundamental roles in defining individual drought responses/impacts, dictating the fate of single trees under global-change-type droughts.

## Introduction

Recent trends towards more frequent anomalous heat and drought waves in broad areas around the world have been suggested to arise from ongoing climate warming (Kumar *et al*., 2013) and have already caused vegetation shifts and forest decline in several biomes (Mueller *et al*., 2005; Allen *et al*., 2010; Nardini *et al*., 2013; Gaylord *et al*., 2015; Hember *et al*., 2017). Considering that climate models forecast increasing intensity/severity of extreme drought events over the next decades, there is an increasing concern about potential impacts on tree species’ survival, regional land cover, ecosystem services and functions.

Heat and drought negatively influence vegetation through effects on water and carbon metabolism (McDowell *et al*., 2011; Reinhardt *et al*., 2015; Salmon *et al*., 2015; Savi *et al*., 2016), and water stress is a common cause of tree decline (Anderegg *et al*., 2013; Nardini *et al*., 2014a). As soil water availability decreases, water potential in xylem conduits (Ψ_xyl_) decreases (Tyree and Zimmermann, 2002), reducing the safety margins against embolism formation (Markesteijn *et al*., 2011; Johnson *et al*., 2012). When species-specific critical Ψ_xyl_ thresholds are surpassed, the capacity of the xylem to supply water becomes insufficient to sustain transpiration (Tyree and Ewers, 1991), and trees may eventually succumb to catastrophic hydraulic failure (Nardini *et al*., 2013).

Heat and drought may also impact the carbon status of trees (Galiano *et al*., 2012; Poyatos *et al*., 2013), as the rate of non-structural carbohydrate (NSC) consumption for metabolism maintenance is correlated with temperature and water status (Amthor, 2000). Furthermore, low water availability leads to stomatal closure and reduced CO_2_ uptake which, coupled to depletion of NSC, can induce negative carbon balance and ‘starvation’ (Allen *et al*., 2010; Sevanto *et al*., 2014; McDowell *et al*., 2016; Nardini *et al*., 2016). Chronic impairment of water transport and carbon balance reduces tree vigor, predisposing plants to biotic attacks that may further impact tree health (Aguadé *et al*., 2015).

Recent experiments have addressed drought-induced tree decline by considering hydraulic failure and carbon starvation as interconnected processes (Aguadé *et al*., 2015; Nardini *et al*., 2016; Savi *et al*., 2016; Yoshimura *et al*., 2016; Petrucco *et al*., 2017; Tomasella *et al*., 2017). It has been suggested that species with a more isohydric response to drought (stomatal control, avoidance of low Ψ_xyl_, conservative water use) might be more exposed to the risk of carbon starvation, while anisohydric plants (maintenance of gas exchange, tolerance of low Ψ_xyl_) can succumb to extreme water shortage because of hydraulic failure (Allen *et al*., 2010; Nardini *et al*., 2014a). However, woody plants exhibit a continuum of hydraulic strategies, rather than a clear distinction between two contrasting alternatives (Johnson *et al*., 2012; Adhikari and White, 2014; Sevanto *et al*., 2014). Furthermore, intraspecific variability in water-use strategies and drought response is not negligible (Poyatos *et al*., 2013; Hentschel *et al*., 2014; Nardini *et al*., 2014b; Garcia-Forner *et al*., 2016; Petrucco *et al*., 2017). With retrospective measurements on mature trees after an extreme drought, Gaylord *et al*. (2015) found a significantly greater frequency of aspirated tracheid pits in dead *Pinus edulis* trees compared to living ones, suggesting their higher vulnerability to embolism formation. Moreover, a significant difference in wood δ^13^C between health classes suggested lower stomatal conductance and greater carbon limitation in declining trees (Gaylord *et al*., 2015). Opposite trends were found by Salmon *et al*. (2015), reporting higher stomatal conductance, transpiration and photosynthetic rates in declining individuals of *Pinus sylvestris* compared to symptom-less trees. However, despite the relatively anisohydric and isohydric response to drought of defoliated and healthy individuals, respectively, few differences were observed in NSC content (Salmon *et al*., 2015). On the other hand, no differences in plant water status and vulnerability to embolism were observed when comparing healthy and declining Scots pine during a dry and warm summer. However, defoliated trees had lower NSC content before, during and after drought in both above- and below-ground organs (Aguadé *et al*., 2015). Overall, intra-population differences in drought-induced tree mortality are difficult to interpret due to possible phenotypic differences in physiological responses, anatomical adaptations and genetic variability of different conspecific individuals (Corcuera *et al*., 2011; López *et al*., 2016), coupled to complex microclimatic and edaphic gradients.

The unusually warm spring–summer droughts that occurred in 2003 and 2012 in southern Europe led to region-wide forest decline (Rebetez *et al*., 2006; Vennetier *et al*., 2007; Nardini *et al*., 2013; Aguadé *et al*., 2015). Petrucco *et al*. (2017) reported widespread dieback of *Pinus nigra* in a karstic region characterized by shallow and highly permeable soils, apparently triggered by the anomalous drought that occurred in the area in 2012 (Nardini *et al*., 2013). Starting from winter–spring 2013, declining and symptom-less individuals were standing next to each other. Dendrochronological measurements coupled with tree-ring carbon and oxygen isotopic composition analysis in differentially affected trees revealed that declining trees had higher growth rates than healthy ones at the beginning of the 20th century, but their growth was reduced after the dry summers of 2003 and 2012. Moreover, results suggested that the two groups adopted different hydraulic strategies under drought, i.e. preferentially isohydric and anisohydric in healthy and declining individuals, respectively (Petrucco *et al*., 2017).

Here, we report measurements of water status and carbon stores of *P. nigra* trees in the years following the extreme drought of 2012. We aimed to investigate post-drought legacies on water and carbon metabolism of declining and healthy trees, as well as possible differences in rooting patterns and/or exploitation of soil water. Hydraulic measurements and anatomical analyses of stem segments were used to highlight different hydraulic efficiency/safety. Based on Petrucco *et al*. (2017), we also hypothesized declining trees to show a more anisohydric strategy and reduced carbon reserves. Finally, we hypothesized that different drought responses might arise from genetic differences due to possible different provenances of seeds used for reforestation.

## Materials and methods

### Study site and plant material

The study was carried out in the Bosco Bazzoni woodland (Trieste, 45° 37.8′ N, 13° 51.7′ E, 380 m a.s.l.). The site is located in the Classical Karst, the highly permeable limestone plateau extending across southwestern Slovenia and northeastern Italy. The pinewood originated from repeated reforestation activities dating back since late 1800s (Sfregola, 2017). The dominant tree species is *P. nigra* J.F. Arnold subsp. *nigra*, while the understory comprises small-sized trees and shrubs like *Cotinus coggygria* Scop., *Fraxinus ornus* L. and *Prunus mahaleb* L. *P. nigra* (Black pine) is a sun-loving species growing on limestone cliffs, and it has been largely exploited for reforestation of arid and rocky habitats (Tutin *et al*., 1964). The climate of the study area is transitional between sub-Mediterranean and prealpine continental. Average annual temperature and rainfalls are ~12.9°C and 1385 mm, respectively (www.osmer.fvg.it; 1992–2016). In 2003 and 2012 the area experienced anomalous summer drought and heat waves. In both episodes, air temperatures in August averaged ~26°C vs 22.5°C of the reference mean, while precipitations during January–August were ~50% less than the average.

Measurements were performed over the period 2014–2016 on five individuals showing dieback and desiccation in more than 50% of their crown (D), as visually estimated by four different observers to reduce possible subjective errors. These were compared to five individuals with still green and apparently healthy crowns (H). D and H trees (height of ~10 m, 30–50 years old) were intermixed with no apparent spatial pattern. Samples collection and measurements were performed on south-exposed branches, at breast height. For genetic analyses (see below), a higher number of D and H trees was sampled.

### Hydraulic measurements

To verify possible differences in terms of stem hydraulic conductivity and resistance to drought-induced xylem dysfunction, in spring 2014 hydraulic vulnerability curves (VCs) were measured with the bench dehydration technique. About 1.5 m long branches were collected from five trees per group early in the morning, re-cut under water, covered with a plastic bag, transported to the laboratory and rehydrated with their cut end immersed in water to favour rehydration (Trifilò *et al*., 2014). Branches sampled from D trees had no dieback, but chlorotic needles. After 24 h, branches were left air dehydrating in the laboratory. At different time intervals, three apical shoots were wrapped in cling film and the branch inserted in a plastic bag. After 30 min of equilibration, Ψ_xyl_ was measured on detached brachiblasts with a pressure chamber (mod. 1505D, PMS Instruments, OR, USA). A stem segment (5–6 years old) was re-cut under water by progressively trimming 2 cm slices at both sides, until obtaining a 4 cm long segment (Venturas *et al*., 2015). Segments were debarked, trimmed and connected to a hydraulic apparatus (Xyl’em, Bronkhorst, Montigny-Les-Corneilles, France). A low pressure perfusion (*P* = 6 kPa) with a 10 mM KCl solution allowed to measure native sample hydraulic conductance (K). The length (l) and transverse xylem area (A_xyl_) of the segment were measured and stem-specific hydraulic conductivity (K_s_) was calculated as (K × l)/A_xyl_. Hence, plots of K_s_ vs Ψ_xyl_ (VCs) were constructed (Hacke *et al*., 2015; Schreiber *et al*., 2016) and a sigmoidal curve model was fitted to the data. Native embolism was not removed, because flushing samples at high pressure resulted in no change or decreased K_s_, as also reported by Froux *et al*. (2002).

Additional experiments were carried out in spring 2016, when relative water loss (RWL) curves of five branches per group (see above) were measured (Rosner *et al*., 2008). Branches were re-cut to a final length of 15 cm, debarked and saturated in distilled and degassed water under partial vacuum for 24 h. After determination of saturated mass (SM), overpressure was applied (*P* = 0.5 MPa, 1 min) to the sample inserted in a double-ended pressure chamber (PMS Instruments, OR, USA) and the stem re-weighed (FM). The treatment was repeated by increasing P in steps of 0.5 or 1 MPa until maximum *P* = 6 MPa. The samples were oven-dried at 103°C, their DM recorded and RWL calculated as [1-((FM-DM)/(SM-DM))] × 100.

### Anatomical measurements and calculation of theoretical hydraulic conductivity

In spring 2014, 10 branches were sampled from H and D individuals and stem segments, similar to those used for hydraulic analyses, were prepared. These samples were used to measure basic wood density (D_w_) as a proxy for drought tolerance (Rosner *et al*., 2008; Markesteijn *et al*., 2011; Trifilò *et al*., 2015). Anatomical analyses were also performed to highlight differences between experimental groups related to post-drought health status. The anatomical characteristics of annual rings formed before the drought event, i.e. under sufficient water supply, were expected to provide information on potential predisposition to drought sensitivity (Rosner *et al*., 2016).

Stem segments were rehydrated overnight, debarked and their fresh volume (V) was measured (Hughes, 2005). Sample mass (DM) was obtained after complete drying (24 h at 103°C), and D_w_ was calculated as DM/V. Samples were softened in a glycerin-ethanol-distilled water solution (1:1:1). After 4 days, 20 μm thick stem cross sections were obtained using a sliding microtome (Reichert-Jung, Optische Werke AG Wien, Austria) and stained with safranin. Images were acquired with a digital camera (Leica DFC 290, Wetzlar, Germany) connected to a microscope (Leica DM 5500B, Wetzlar, Germany) and analysed with ImageJ (https://imagej.nih.gov/ij/). Considering the last three complete annual rings (2011–2013), the following parameters were quantified: ring width and annual ring area, percentage of latewood, tracheid diameter (d), tracheid double wall thickness (t), the square of the thickness-to-span ratio (t/b)^2^, tracheid density (T_n_) and the hydraulic mean diameter (D_h_), calculated as (i) Σd^5^/Σd^4^ (Kolb and Sperry, 1999; Hacke *et al*., 2001; Petit *et al*., 2016) and (ii) (Σd^4^/N)^0.25^ (Tyree and Zimmermann, 2002; Scholtz *et al*., 2013), when N is the total number of conduits measured. About 200 individual tracheids were measured on each branch. Using D_h_ calculated as suggested by Tyree and Zimmermann (2002) in the Hagen–Poiseuille equation, an estimate of hydraulic conductivity (K_theor_) for each growth ring was calculated according to Gonzalez-Benecke *et al*. (2010) as T_n_ × π × D_h_^4^/128 η, where η is the viscosity of water (8.9 × 10^−10^ MPa s).

### Monitoring of plant water status and hydraulic strategies

To highlight possible differences in water status between H and D trees, leaf water potential and conductance to water vapour (g_L_) were measured on two sunny days in spring (June) and summer (July) 2015. Pre-dawn (Ψ_pd_) and minimum (Ψ_min_) water potential were measured on brachiblasts detached at 5.00 am and 13.00 am, respectively, from each of the 10 individuals. Samples were wrapped in cling film, transported to the laboratory in a cool bag and measured with the pressure chamber within 2 h from sampling. At midday, g_L_ was measured on at least two brachiblasts per individual with a porometer (LI-1600, Li-Cor Inc., NE, USA). During all measurements, ambient air temperature (T_air_, 25.2 ± 0.1 and 38.3 ± 0.3°C in June and July, respectively), relative humidity (RH, 22.6 ± 0.2 and 20.4 ± 0.3%) and photosynthetic photon flux density (PPFD, 1562 ± 80 and 1581 ± 45 μmol m^−2^ s^−1^) were recorded. Maximum vapour pressure deficit (VPD) was 2.5 kPa and 5.4 kPa in spring and summer, respectively.

To check for eventual differences in stomatal responses to water stress in H and D trees (anisohydry vs isohydry), the simultaneous decrease of Ψ_leaf_ and g_L_ was monitored on air-dehydrating detached branches. In July 2015, five branches were sampled (before 9.00 am) from each group and rehydrated as described above. At midday, the plastic bag was removed and the branches exposed to sun irradiance (PPFD = 1623 ± 61 μmol m^−2^ s^−1^, VPD = 3.6 kPa) while maintaining their cut end in water. After 30 min, g_L_ and Ψ_leaf_ were measured on at least two brachiblasts per branch. Branches were then removed from the water and g_L_ and Ψ_leaf_ were re-measured every 20 min. Measurements continued for 2–3 h until g_L_ values close to zero were recorded (Ψ_leaf_ about −2.5 MPa). Since the recorded g_Lmax_ was slightly different in the different branches, data were normalized by calculating the relative leaf conductance to water vapour (g_L_REL_): g_L_/g_Lmax_, and plotted vs Ψ_leaf_.

### Isotopic composition of xylem sap

To verify eventual heterogeneity of soil water sources accessed by H and D trees, oxygen isotopic composition of xylem sap was measured (Ehleringer and Dawson, 1992). In spring and summer 2015, on the same dates selected for water status measurements, xylem sap samples were collected from the same individuals used for other experiments. Four- to six-year-old branches were detached at midday, quickly debarked, cut in small pieces and enclosed in sealed plastic bags. Samples were transported to the laboratory in a cool bag and stored at −20°C. Xylem sap was extracted with a cryogenic vacuum distillation method (West *et al*., 2006). Between mid-June and mid-July, rainfall occurring in the study site was also collected using a rain gauge containing a film of paraffin oil to avoid evaporation. The oxygen isotope composition (δ^18^O) of samples was measured with an isotope ratio mass spectrometer (Delta Plus Advantage, Thermo Fisher Scientific, MA, USA; see also Nardini *et al*., 2016).

### NSC contents

To highlight eventual differences in post-drought NSC reserves, soluble sugars (glucose, fructose and sucrose) and starch concentrations were measured on 3- to 5-year-old stem segments detached from H and D trees (one stem segment per tree, five trees per group) on the dates selected for water status measurements. Samples were transported to the laboratory in a cool bag, microwaved (700 W, 3 min; within 1 h after sampling), oven-dried at 70°C and kept frozen until analysis. Samples were pulverized (particle size <0.15 mm), dividing bark and wood, and 15 ± 1 mg of material was transferred in a 1.5 ml Eppendorf vial. NSC extraction and analysis followed the enzymatic method standardized by Quentin *et al*. (2015) adapted to low amounts of material (Savi *et al*., 2016). Samples were suspended in 1 ml of 80% ethanol solution for three times, the supernatant was used for soluble sugars measurement, while the pellet was re-suspended in 1 ml of Acetate buffer (0.4 M NaCH3COO, pH = 4.6) and directed to starch evaluation. For glucose measurement, 5–20 ml of supernatant were transferred in a cuvette with 2 ml final volume of essay buffer solution solution (Tris-HCl with MgCl2 5 mM, NADP+ 125 μM and MgATP 1 mM, at 25°C) and placed in a spectrofluorimeter (LS50B Luminescence Spectometer, Perkin-Elmer, MA, USA). The reaction was conducted by adding 2 U of both glucose-6-phosphate dehydrogenase and hexokinase. When the enzymatic kinetic due to gluconolactone production reached steady state, the evaluation of fructose was obtained adding in the same cuvette 3 U of phosphoglucose isomerase, to convert fructose-6-phosphate produced with hexokinase in glucose-6-phosphate. For sucrose analysis, 100 ml of the supernatant were placed in a 1.5 ml Eppendorf tube with 300 ml of acetate buffer with of 25 U of invertase to break down sucrose into fructose and glucose. The tubes were kept at 55°C for 30 min, then 20 ml were processed as described above. For starch digestion we performed an overnight procedure at 55°C using 100 U of α-amylase and 25 U of amylogucosidase per sample. To prevent further degradation, the samples were boiled for 3 min. For analyses, 10 μl of final supernatant were transferred in a cuvette with 2 ml final volume of essay buffer. The starch digestion and spectrofluorimeter analysis was also performed with known amounts of Amylose to obtain a calibration curve. The final concentration of starch in the sample was then expressed as % dry mass (% DM).

### Genetic analysis

Because the pine woodland under study results from reforestation activities, we verified the eventual genotypic basis for different responses to drought between D and H trees, as a possible consequence of different seeds provenances. Three chloroplast microsatellites loci [simple sequence repeats (SSRs)] were analysed (Naydenov *et al*., 2006). In April 2015, green needles of 50 trees per experimental category were collected from trees belonging to different age classes (20–100 years old), grinded in liquid nitrogen and stored frozen. Total DNA was extracted with the E.Z.N.A. kit (Plant DNA kit, Omega Bio-tek Inc, Norcross, GA, USA), quantified with a spectrophotometer (NanoDrop, Thermo Fisher Scientific, MA, USA) and three plastome microsatellites loci (Pt30204, Pt71936, Pt45002) were amplified with fluorescently labelled primers (Naydenov *et al*., 2006). Amplicons were resolved on agarose gel to verify amplification efficiency and quality and finally molecular weights were analysed using the ABI 3130 capillary sequencer with a ROX-labelled size standard (ABI 3130 Genetic Analyzer, Applied Biosystem, CA, USA). As a control, the same loci were also analysed in needles of three individuals of *Pinus halepensis*.

### Statistical analysis

Data were analysed using SigmaStat v. 2.03 (SPSS Inc.) and R (R i386 3.2.5). Data normality and homoscedascity were assessed and statistically significant differences were highlighted by Student’s *t*-test, two- and three-way ANOVA (analysis of variance) (*P* < 0.05). An ANCOVA (analysis of covariance) was applied to test differences in the response to drought of H and D trees. The SSRs data were analysed with the software STRUCTURE. The most likely number of clusters (K) was estimated using the complementary software Structure Harvester v 0.6.94 (Pritchard *et al*., 2000).

## Results

**Figure 1:**
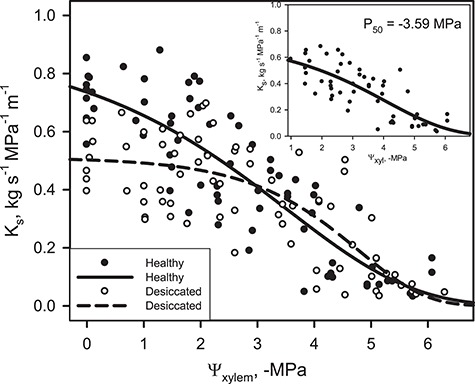
Vulnerability curves (VCs) reporting the relationship between stem-specific hydraulic conductivity (K_s_) and xylem water potential (Ψ_xyl_), as measured for healthy (H, closed circles, solid line) and desiccated (D, open circles, dashed line) *P. nigra* trees. The sigmoidal regressions are also reported. The Ψ_xyl_ inducing 20 (P_20_) and 50 (P_50_) % loss of K_s_ were −1.67 and −3.24 MPa, and −1.42 and −3.63 MPa for H and D group, respectively (fit-PLC, Duursma and Choat, 2016). The insets show the VC of H trees based on K_s_ data that were within the observable range for both populations (0.03–0.68 kg s^−1^ MPa^−1^ m^−1^).

Hydraulic VCs of D and H trees (Fig. 1) were based on 71 and 74 hydraulic measurements, respectively, and the reference parameters P_20_ and P_50_ (Ψ_xyl_ inducing 20 and 50% loss of K_s_) were derived from VCs using the r-package fit-PLC (Duursma and Choat, 2016). The P_20_ resulted slightly more negative in H than in D individuals (−1.7 vs 1.4 MPa), while P_50_ was lower in D ones (D = −3.6 MPa, H = −3.2 MPa). However, both parameters were not significantly different between the two groups of trees, as indicated by the overlapping 95% confidence intervals (Table S1). The maximum stem-specific hydraulic conductivity (K_max_), calculated as the average K_s_ at Ψ_xyl_ > −0.5 MPa, was significantly lower (*P* < 0.001) in D trees (0.52 ± 0.04 kg s^−1^ MPa^−1^ m^−1^) than in H ones (0.72 ± 0.03 kg s^−1^ MPa^−1^ m^−1^). The inset in Fig. 1 reports the VC of H trees based on K_s_ values within the observable range for both populations (0.03–0.68 kg s^−1^ MPa^−1^ m^−1^). In this case, the recalculated P_50_ for H trees is shifted towards more negative values (−3.6 MPa). The two experimental groups did not differ in terms of basic wood density, which averaged 0.46 ± 0.02 g cm^−3^ and 0.46 ± 0.01 g cm^−3^ in H and D, respectively. No significant differences in terms of annual ring area, tracheid diameter, hydraulic mean diameter and theoretical hydraulic conductivity were recorded between experimental groups and among the three annual rings analysed (Table 1; Table S2). Annual tree growth, estimated by ring width, was similar in D and H trees, but significantly lower in 2013 than in 2011. H trees showed higher values of (t/b)^2^ than D ones (0.17 ± 0.01 vs 0.13 ± 0.01, three annual rings considered). A slight reduction of (t/b)^2^ emerged when comparing 2011 and 2013, but the difference among years was not statistically significant. However, in 2012 (the year of the drought event), D trees had significantly lower (t/b)^2^ than H trees (Table 1, Student’s *t*-test).

**Table 1:** Ring width, annual ring area, percentage of latewood, tracheid diameter (d), tracheid wall thickness (t), thickness-to-span ratio, tracheid density, hydraulic mean diameters (D_h_) and theoretical hydraulic conductivity (K_theor_) measured for healthy (H) and desiccated (D) individuals in the last tree annual rings (2011–2013)

	**Healthy**			**Desiccated**		
	**2011**	**2012**	**2013**	**2011**	**2012**	**2013**
Ring width, mm	**0.29 ± 0.05** ^**a**^	**0.20 ± 0.04** ^**ab**^	**0.17 ± 0.03** ^**b**^	**0.33 ± 0.06** ^**a**^	**0.23 ± 0.08** ^**ab**^	**0.15 ± 0.02** ^**b**^
Ring area, mm^2^	4.6 ± 0.7	3.3 ± 0.9	2.9 ± 0.7	4.7 ± 0.8	4.2 ± 1.24	2.9 ± 0.7
Late wood, %	27.7 ± 4.4	24.1 ± 5.2	28.2 ± 4.9	27.6 ± 6.6	18.4 ± 2.8	28.8 ± 5.8
Tracheid diameter, μm	12.0 ± 0.4	12.4 ± 0.3	12.2 ± 0.3	12.4 ± 0.3	12.7 ± 0.3	12.1 ± 0.5
Wall thickness, μm	4.0 ± 0.3	3.8 ± 0.2	3.6 ± 0.2	3.7 ± 0.2	3.4 ± 0.4	3.7 ± 0.3
(t/b)^2^	**0.19 ± 0.03** ^**A**^	**0.17 ± 0.01** ^**A**^	**0.15 ± 0.02** ^**A**^	**0.14 ± 0.03** ^**B**^	**0.11 ± 0.02** ^**B**^	**0.13 ± 0.01** ^**B**^
Tracheid density, mm^−2^	3739 ± 83	3687 ± 231	3850 ± 293	3868 ± 49	3979 ± 106	3855 ± 275
D_h_ (Kolb and Sperry, 1999), μm	15.7 ± 0.6	16.3 ± 0.3	15.6 ± 0.4	17.1 ± 0.5	16.2 ± 0.7	15.8 ± 0.7
D_h_ (Tyree and Zimmermann, 2002), μm	13.2 ± 0.4	13.8 ± 0.2	13.1 ± 0.3	13.9 ± 0.3	13.6 ± 0.4	13.1 ± 0.4
K_theor_, kg s^−1^ MPa^−1^ m^−1^	3.2 ± 0.2	3.7 ± 0.1	3.1 ± 0.1	4.0 ± 0.2	3.8 ± 0.3	3.2 ± 0.2

Values with significant differences are reported in bold. Mean ± SEM are reported. Upper-case letters indicate statistically significant difference (*P* < 0.05) between health classes (Factor I), while lower-case letters indicate statistically significant difference among years (Factor II), as tested using two-way ANOVA. No statistically significant interaction between factors was observed.

Significant differences between experimental groups emerged from RWL curves (Fig. S1). RWL was found to be significantly higher (*P* < 0.05) in D compared to H individuals at four different overpressures. The reference parameter Ψ_RWL30_, i.e. the overpressure inducing 30% RWL, was 3.54 and 4.14 MPa for D and H trees, respectively.

In spring and summer, under respectively high and low soil water availability, Ψ_pd_ averaged −0.45 MPa and −1.37 MPa in both experimental groups (Table 2). The Ψ_min_ dropped to about −1.00 MPa in spring and reached −1.70 MPa in summer. A non-significant (*P* = 0.1) trend towards more negative Ψ_min_ in D than in H trees was observed, consistent with ~30% higher g_L_ values measured in spring in the former group compared to the latter (213 ± 26 vs 160 ± 13 mmol m^−2^ s^−1^, *P* = 0.1).

**Table 2:** Pre-dawn (Ψ_pd_) and minimum water potential (Ψ_min_) and leaf conductance to water vapour (g_L_) measured in healthy (H) and desiccated (D) trees in June (a) and July (b) 2015

	**Healthy**	**Desiccated**
**(a)**	**17 June 2015**	
Ψ_pd_, −MPa	0.44 ± 0.01	0.49 ± 0.04
Ψ_min_, −MPa	0.96 ± 0.03	1.08 ± 0.06
g_L_, mmol m^−2^ s^−1^	160.2 ± 12.8	213.2 ± 25.7
**(b)**	**22 July 2015**	
Ψ_pd_, −MPa	1.38 ± 0.03	1.35 ± 0.04
Ψ_min_, −MPa	1.69 ± 0.03	1.73 ± 0.05
g_L_, mmol m^−2^ s^−1^	28.4 ± 11.8	31.3 ± 3.09

Mean ± SEM are reported.

**Figure 2:**
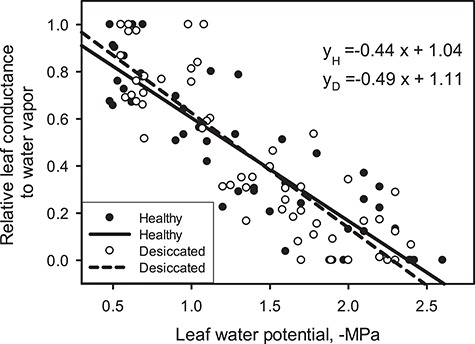
Relationship between relative leaf conductance to water vapour (g_L_REL_), as measured in H (closed circles, solid line) and D (open circles, dashed line) branches at progressively lower leaf water potential (Ψ_leaf_). Coefficients of the linear regressions are also reported.

Figure 2 reports changes in relative leaf conductance to water vapour (g_L_REL_) measured in H and D branches as a function of Ψ_leaf_. D and H trees did not differ in terms of maximum g_L_ measured in fully hydrated branches exposed to sun, which averaged 433 ± 30 mmol m^−2^ s^−1^. The ANCOVA test did not highlight statistically significant differences in the response of g_L_ to Ψ_leaf_ between the two categories.

**Figure 3:**
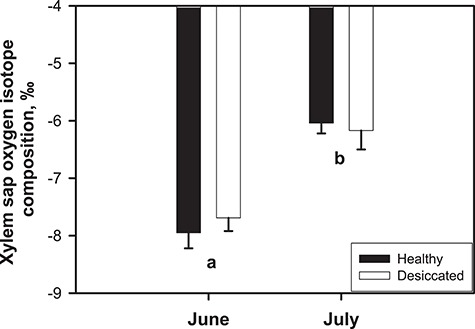
Oxygen isotopic composition of xylem sap (δ^18^O) extracted from branches of healthy (H, black columns) and desiccated (D, grey columns) individuals in June and July 2015. Mean ± standard error of the mean (SEM) are reported. Lower-case letters denote a significant difference between sampling seasons (June vs July), while differences between D and H trees were not significant (two-way ANOVA). No statistically significant interaction between factors was observed.

The oxygen isotopic composition of xylem sap extracted from H and D stems in June averaged −8.0 ± 0.3‰ and −7.7 ± 0.2‰, respectively (Fig. 3). In July, the δ^18^O significantly increased (less negative values) to about −6.1‰, but differences between experimental groups were not statistically significant (two-way ANOVA). The δ^18^O value of summer rainfalls averaged −4.6‰.

**Figure 4:**
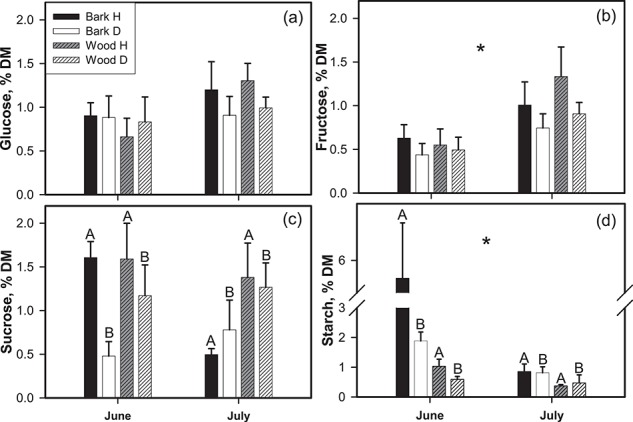
Glucose (a), fructose (b), sucrose (c) and starch (d) concentration measured in bark and wood of H (black and grey dashed columns, respectively) and D (white and white dashed columns, respectively) trees in June and July 2015. Mean ± SEM are reported. Upper-case letters and asterisks indicate statistically significant difference (*P* < 0.05) between health classes (Factor I) and growing seasons (Factor III), respectively. For sucrose and starch statistically significant differences between wood and bark tissue (Factor II) was also observed. Complete output of the three-way ANOVA in Table S2

Figure 4 shows NSC concentrations measured in bark and wood of H and D trees. The output of the three-way ANOVA is reported in Table S2. No statistically significant differences in glucose and fructose contents were observed between health classes and tissues. While glucose values were fundamentally similar in June and July (0.8% and 1% DM, respectively), a significant increase of fructose (by ~100%) was observed at the peak of drought. Starch content ranged from 0.37 ± 0.04% DM to 5.6 ± 1.4% DM in wood and bark of H trees in summer and spring, respectively. Overall, starch concentration was higher in spring than in summer by ~200%, and markedly higher in bark than in wood. Furthermore, sucrose and starch content were by ~40% and 120% higher in H trees compared to D, respectively. The statistically significant seasonal drop of starch content was more pronounced in H trees than in D ones.

Genetic polymorphism data from three chloroplast microsatellites loci were obtained from 50 different individuals per experimental group (Table S3). The size range was 136–142 bp, 142–146 bp and 162–165 bp for the loci Pt30204, Pt71936 and Pt45002, respectively, i.e. very similar to those found in the reference paper for the same species (Naydenov *et al*., 2006). The sizes found for *P. halepensis* (control species) were 134 bp, 148 bp and 159 bp for the loci Pt30204, Pt71936 and Pt45002, respectively. Using STRUCTURE analysis, we estimated the potential number of populations (K) to which the individuals could be attributed, without being assigned a priori to hypothetical populations. The analysis indicated that the probability of *K* = 1 was greater than that of *K* = 2. To validate the hypothesis that there is no population structure, we further performed a supervisioned clustering with *K* = 2. Figure S2 shows the probability of assignment of each individual to two putative populations.

## Discussion

Anomalous drought and heat waves have produced large-scale impacts on forest ecosystems (Matusick *et al*., 2013; Aguadé *et al*., 2015; Hember *et al*., 2017). One year after the 2012 severe drought, several Black pines suffered extensive crown dieback. On the basis of visual assessments performed in spring 2014, 2015 and 2016 no signs of recovery could be detected in D individuals, while H plants showed still completely healthy crowns. Our retrospective analyses revealed both similarities and subtle differences between declining and symptom-less trees.

The analysis of VCs yielded a P_50_ of about −3.2 MPa for H trees, in accordance with published values for the same species (Froux *et al*., 2002; Martínez-Vilalta and Piñol, 2002; Hacke *et al*., 2001) and within the range reported for other *Pinus* species (P_50_ varying from −2.3 MPa to −7.0 MPa). Hence, Black pine appears as a species relatively vulnerable to drought-induced xylem dysfunction (Hacke *et al*., 2001; Lamy *et al*., 2011; Sáenz-Romero *et al*., 2013; Battipaglia *et al*., 2016). Hydraulic VCs also suggested a slightly higher resistance against drought-induced embolism of D individuals, which displayed ~0.4 MPa lower (more negative) P_50_ than that recorded in H trees. Although not significant, this is an opposite trend compared to data reported in recent studies (Anderegg *et al*., 2013; Nardini *et al*., 2014b). While the native hydraulic conductivity (K_s_) of H trees was in agreement with previously published values (Froux *et al*., 2002), D trees showed a significantly lower K_s_, as a possible consequence of embolism accumulated during the drought period. In fact, when the VC of H trees was recalculated based only on values of K_s_ that were within the observable range for D trees, the interpolated P_50_ was nearly identical in the two groups (see inset in Fig. 1).

Plant hydraulic performance is influenced by xylem anatomy (Hacke *et al*., 2001; Tyree and Zimmermann, 2002; Scholz *et al*., 2013; Petit *et al*., 2016), with wider conduits leading to higher K_s_. In particular, hydraulically weighted tracheid diameter (Tyree and Zimmermann, 2002) is linearly related to K_max_ in pine species (Gonzalez-Benecke *et al*., 2010). Interestingly, we did not observe anatomical differences between D and H trees (Table 1) that could account for recorded differences in K_s_. Ring width and area, tracheid diameter, hydraulic diameters, as well as theoretical hydraulic conductivity, were similar in H and D individuals in the outermost annual rings. H individuals had (t/b)^2^ ~30% higher than D ones (0.17 ± 0.01 vs 0.13 ± 0.01, pooled for the three outermost rings), suggesting higher conduit reinforcement and consequently higher safety towards tracheid collapse under negative pressure in H trees. Since (t/b)^2^ has been correlated with the resistance against embolism formation (Hacke *et al*., 2001; Willson and Jackson, 2006; Rosner *et al*., 2016), this finding suggests that D trees were more vulnerable to drought-induced loss of water transport efficiency. This is also supported by RWL curves, showing that D trees have a less negative xylem pressure threshold inducing embolism formation compared to H trees and lose significantly larger water volumes at similar Ψ_xyl_ (Fig. S1; Table S1). The overpressure resulting in 50% RWL is considered a good proxy for hydraulic vulnerability (Rosner *et al*., 2008). In our study, Ψ_RWL50_ was reached at very high overpressure (~6 MPa) but, interestingly, interpolating Ψ_RWL30_ from the fitted models resulted in a difference of 0.6 MPa between D and H trees (3.54 MPa vs 4.14 MPa, respectively).

Overall, the results of hydraulic and anatomical analyses suggest that the observed difference in terms of K_s_ was a legacy of the drought event that produced a differential impact on the two groups of trees, so that tracheids of D stems were partially embolized and not conductive when hydraulic measurements were performed. This residual embolism level was apparently not recovered during night-time branch rehydration prior to hydraulic measurements. As a consequence, the K_s_ of D trees was significantly lower at any given Ψ_xyl_ and the VC was ‘shifted’ towards more negative Ψ_xyl_ values with respect to that of H trees (Hacke *et al*., 2015). The reduced efficiency of water delivery to foliage, consequent to the accumulated embolism, might provide an explanation for the observed partial desiccation of the crown in D individuals.

The lower cell wall reinforcement of D trees revealed limited carbon investments in xylem safety in declining individuals, suggesting limitations in terms of NSC availability as a consequence of reduced photosynthetic area due to defoliation, stomatal aperture and limitation of carbon fixation (Poyatos *et al*., 2013). NSC analyses confirmed that carbon stores were reduced in D trees (Fig. 4; Table S2). The increase of glucose and fructose concentration observed in July in both groups suggests that H and D trees likely adopted the same strategy of NSC mobilization to cope with seasonal drought. The increase of soluble sugars can arise as a consequence of both photosynthetic fixation and degradation of NSC (consistent with observed starch depletion). Intriguingly, sucrose and starch showed a different behaviour in H and D plants with the latter displaying significantly lower (by ~50%) starch concentration compared to the former. This difference was evident in spring, while values of starch concentration were more similar in summer, when the larger reserves in H trees had been depleted already. The significant drop in starch supports the hypothesis of its mobilization under drought, likely leading to higher sugar availability in H than in D trees (Δ starch 5.37 and 1.19% DM, respectively) providing energy to support growth and metabolism. Higher concentrations of NSC in healthy pine trees compared to desiccated ones, and an increase of soluble sugars under drought have been already reported, suggesting that both defoliation and prolonged periods of near complete stomatal closure contribute to reduce NSC in trees (Galiano *et al*., 2012; Poyatos *et al*., 2013; Aguadé *et al*., 2015; Vilela *et al*., 2016).

Our data suggest that the decline of some *P. nigra* individuals arise as a consequence of xylem embolism triggered by drought stress, coupled to impending carbon starvation (Kono *et al*., 2019). Considering that the woodland under study results from reforestation, we hypothesized that the differential drought impact on individuals could be linked to different origins of seeds/seedlings used, also taking into account that *P. nigra* displays high genetic distance between populations (Thiel *et al*., 2012) and considering that provenance-based differences in xylem vulnerability have been reported for different *Pinus* species. However, genetic analysis focused on the plastome did not highlight the presence of distinct populations among sampled individuals. Our analysis was based on the study of only three microsatellites loci, and it was aimed at detecting eventual differences in the geographical origin of seeds used for plant production and afforestation in the late 1800s. On this basis, we cannot exclude the occurrence of other genotypic differences between H and D trees, which might eventually explain their different vulnerabilities and also represent an interesting starting basis for selection of *P. nigra* genotypes better adapted to future climate scenarios. Indeed, previous studies have shown that the variability of vulnerability to drought in different tree species can be larger within populations than between populations (Corcuera *et al*., 2011; Wortemann *et al*., 2011). In order to obtain conclusive evidence on this point in the case of *P. nigra*, a complete genetic analysis based on genome-wide scan methodologies would be needed to reveal differences between the two groups.

Isotopic analyses of xylem sap revealed that D and H trees exploited fundamentally the same water pool. In fact, δ^18^O was about −8‰ in June and −6‰ in July for both groups, suggesting lack of differences in rooting depth (Ehleringer and Dawson, 1992). This does not rule out the possibility that D trees have a less extensive root system or are deficient in some other way in their ability to access and absorb water. A comparison of our data with a recent study focused in an area located <1 km from the pine woodland (Nardini *et al*., 2016) suggests prevalent use of shallow water resources by Black pine in our experimental site.

A recent study by Petrucco *et al*. (2017), performed in the same study site, has reported lower values of δ^13^C in wood cores of D trees compared to H ones, suggesting delayed stomatal closure under drought and more pronounced water-spending behaviour in the former group. In our study, seasonal changes in Ψ_leaf_ and g_L_ indicated that D and H trees did not experience different levels of water stress during the normal seasonal drought that occurred in 2015. This is consistent with findings by Aguadé *et al*. (2015), reporting similar Ψ values in *P. sylvestris* affected by desiccation compared to still healthy individuals, but opposite to results of Hentschel *et al*. (2014) and Salmon *et al*. (2015). However, during spring we recorded a weak and marginally significant trend (*P* = 0.1) towards lower Ψ_min_ (by ~13%) and higher g_L_ (by ~33%) in D trees compared to H ones. These results would support the hypothesis that D trees display a more anisohydric response to drought (Petrucco *et al*., 2017). Considering the reduced photosynthetic area of D trees, their slightly more pronounced stomatal opening might also be interpreted as a compensatory physiological mechanism to maintain an overall positive carbon gain. Similarly, recent studies recorded significantly higher stomatal conductance and net CO_2_ assimilation in defoliated than in non-defoliated Scots pines and Norway spruce, with the former group displaying also significantly lower Ψ_pd_ and Ψ_min_ (Hentschel *et al*., 2014; Salmon *et al*., 2015).

Opposite to previous findings highlighting different responses of canopy stomatal conductance to Ψ changes in defoliated and non-defoliated *P. sylvestris* (Poyatos *et al*., 2013), the g_L_ response to Ψ_leaf_ was very similar in H and D trees, suggesting that stomata responded similarly to leaf dehydration in the two groups. Stomata are known to respond to several different environmental and physiological factors, besides needle water status (Mott and Peak, 2011; McAdam and Brodribb, 2015). Hence, we cannot exclude different intrinsic responsiveness of stomata of H and D trees to other factors such as air temperature, RH, wind and irradiance. In particular, differences in g_L_ response to VPD at relatively constant Ψ_leaf_ values, not tested in our study, might provide an explanation for different hydraulic strategies in H and D trees (McAdam and Brodribb, 2015; Marchin *et al*., 2016).

Our study confirms that drought-induced canopy desiccation is a complex output of interactions between water and carbon metabolism. In fact, differences in the carbon status of *P. nigra* trees after a severe drought can apparently lead to subtle differences in the xylem structure and its efficiency, which may develop into declining tree vigor. These data, coupled to similar conclusions by recent studies (Galiano *et al*., 2012; Salmon *et al*., 2015), suggest that the analysis of species-specific resistance against drought is not sufficient to fully understand and model the phenomenon of drought-induced tree die-off. The assessment of the risk of tree decline, and the development of quantitative and qualitative predictions of species abundance, diversity and richness under climate change scenario, will need to take into account that small and continuous variations in environmental and edaphic characteristics (Davi and Cailleret, 2017), as well as genotypic or phenotypic intraspecific variability (Taeger *et al*., 2013; Stojnić *et al*., 2017) can play fundamental roles in defining individual drought responses/impacts, dictating the fate of single trees under global-change-type droughts.

## Supplementary Material

Supplementary_Information_coz012
