# Supplementary material for: Drought-induced dieback of Pinus nigra: a tale of hydraulic failure and carbon starvation
Source: Conserv Physiol. 2019 May 15;7(1):coz012. doi: 10.1093/conphys/coz012 (PMC6541882; doi:10.1093/conphys/coz012)
Supplement: Supplementary_Information_coz012 [file supplementary_information_coz012.doc]

LEGENDS

Figure S1: Relative water loss curves reporting the relationship between RWL and the applied overpressure (P), as measured for healthy (H, closed circles, solid line) and desiccated (D, open circles, dashed line) *Pinus nigra* trees. Mean values ± SEM are shown. The sigmoidal regressions and P inducing 30% RWL are also reported. * indicates significant differences between experimental groups (P<0.05).

Figure S2: Assignment of individuals to genetic clusters using STRUCTURE with K=2. Each bar represents an individual and the dimensions of the different area are proportional to the genetic relatedness of the individual to the two clusters.

Table S1: P_20_ and P_50_ interpolated from vulnerability curves (VCs) of healthy (H) and desiccated (D) trees and relative 95% confidence intervals estimated from the bootstrap method (r-package fit-PLC, R i386 3.2.5, Duursma and Choat 2016).

Table S2: Summary of the three-way-ANOVA testing the effects of plant health status (H or D, Factor I), tissue (bark or wood, Factor II), and season (June or July, Factor III), and their interaction on glucose, fructose, sucrose, and starch content at the branch scale.

Table S3: Genetic profiles of healthy (H) and desiccated (D) *Pinus nigra* trees analyzed at three plastome microsatellites loci: Pt30204, Pt71936, and Pt45002.

Figure S1


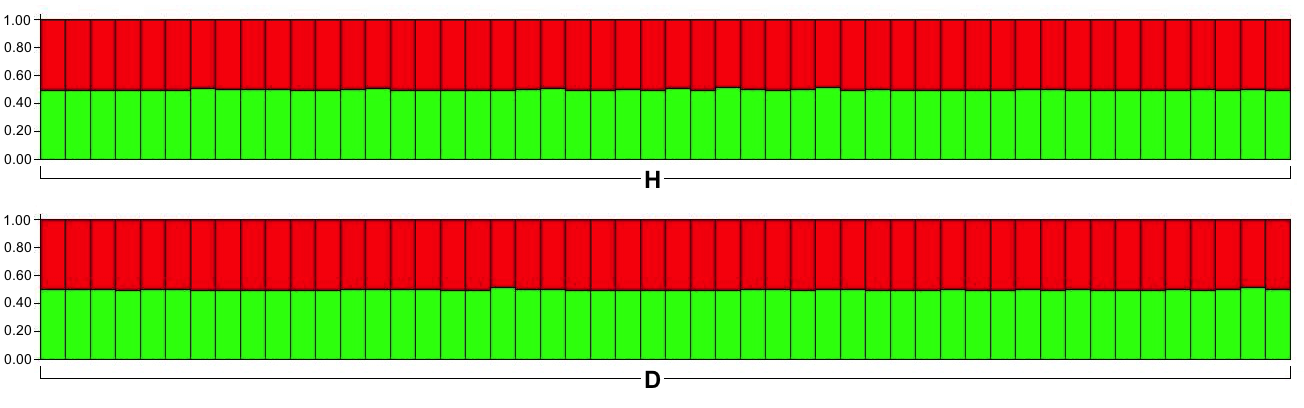


Figure S2

|  | Healthy | | | Desiccated | | |
| --- | --- | --- | --- | --- | --- | --- |
|  | Px, -MPa | 2.50% | 97.50% | Px, -MPa | 2.50% | 97.50% |
| P_20_ | 1.67 | 1.21 | 2.07 | 1.42 | 0.64 | 2.12 |
| P_50_ | 3.24 | 2.95 | 3.54 | 3.63 | 3.09 | 4.38 |

Table S1

| **Glucose, % DW** | ***df*** | ***SS*** | ***MS*** | ***F*** | ***P*** |
| --- | --- | --- | --- | --- | --- |
| **Health status** | 1 | 1.44 | 0.14 | 0.55 | 0.47 |
| **Tissue** | 1 | 0.004 | 0.004 | 0.02 | 0.9 |
| **Season** | 1 | 0.68 | 0.68 | 2.56 | 0.12 |
| **Fac. I × Fac. II** | 1 | 0.03 | 0.03 | 0.09 | 0.76 |
| **Fac. I × Fac. III** | 1 | 0.44 | 0.44 | 1.67 | 0.21 |
| **Fac. II × Fac. III** | 1 | 0.12 | 0.12 | 0.46 | 0.5 |
| **Fac. I × Fac. II × Fac. III** | 1 | 0.04 | 0.04 | 0.14 | 0.71 |
| **Residual** | 32 | 8.45 | 0.26 |  |  |
| **Fructose, % DW** | ***df*** | ***SS*** | ***MS*** | ***F*** | ***P*** |
| **Health status** | 1 | 0.55 | 0.55 | 2.73 | 0.11 |
| **Tissue** | 1 | 0.14 | 0.14 | 0.68 | 0.42 |
| **Season** | 1 | 2.22 | 2.22 | 10.98 | **0.002** |
| **Fac. I × Fac. II** | 1 | 0.001 | 0.001 | 0.003 | 0.96 |
| **Fac. I × Fac. III** | 1 | 0.12 | 0.12 | 0.60 | 0.44 |
| **Fac. II × Fac. III** | 1 | 0.16 | 0.16 | 0.81 | 0.38 |
| **Fac. I × Fac. II × Fac. III** | 1 | 0.06 | 0.06 | 0.28 | 0.6 |
| **Residual** | 32 | 6.46 | 0.20 |  |  |
| **Sucrose, % DW** | ***df*** | ***SS*** | ***MS*** | ***F*** | ***P*** |
| **Health status** | 1 | 2.33 | 2.33 | 4.36 | **0.045** |
| **Tissue** | 1 | 4.63 | 4.63 | 8.67 | **0.006** |
| **Season** | 1 | 0.19 | 0.19 | 0.36 | 0.56 |
| **Fac. I × Fac. II** | 1 | 0.74 | 0.74 | 1.38 | 0.25 |
| **Fac. I × Fac. III** | 1 | 2.78 | 2.78 | 5.20 | **0.03** |
| **Fac. II × Fac. III** | 1 | 0.20 | 0.20 | 0.37 | 0.55 |
| **Fac. I × Fac. II × Fac. III** | 1 | 1.49 | 1.49 | 2.78 | 0.11 |
| **Residual** | 32 | 17.10 | 0.53 |  |  |
| **Starch, % DW** | ***df*** | ***SS*** | ***MS*** | ***F*** | ***P*** |
| **Health status** | 1 | 1.66 | 1.66 | 4.29 | **0.047** |
| **Tissue** | 1 | 12.03 | 12.03 | 31.11 | **<0.001** |
| **Season** | 1 | 11.36 | 11.36 | 29.38 | **<0.001** |
| **Fac. I × Fac. II** | 1 | 0.04 | 0.04 | 0.10 | 0.76 |
| **Fac. I × Fac. III** | 1 | 0.92 | 0.92 | 2.40 | 0.13 |
| **Fac. II × Fac. III** | 1 | 0.90 | 0.90 | 2.33 | 0.14 |
| **Fac. I × Fac. II × Fac. III** | 1 | 0.31 | 0.31 | 0.79 | 0.38 |
| **Residual** | 32 | 12.38 | 0.39 |  |  |

Table S2

| **(a)** | **Pt30204** | **Pt71936** | **Pt45002** |  | **(b)** | **Pt30204** | **Pt71936** | **Pt45002** |
| --- | --- | --- | --- | --- | --- | --- | --- | --- |
| **H1** | 139 | 145 | 164 |  | **D1** | 140 | 144 | 163 |
| **H2** | 141 | 145 | 164 |  | **D2** | 140 | 145 | 163 |
| **H3** | 139 | 144 | 164 |  | **D3** | 140 | 145 | 164 |
| **H4** | 140 | 144 | 164 |  | **D4** | 140 | 144 | 164 |
| **H5** | 139 | 143 | 164 |  | **D5** | 139 | 144 | 163 |
| **H6** | 141 | 143 | 164 |  | **D6** | 142 | 145 | 164 |
| **H7** | 142 | 146 | 164 |  | **D7** | 139 | 143 | 164 |
| **H8** | 140 | 145 | 164 |  | **D8** | 141 | 144 | 164 |
| **H9** | 138 | 144 | 164 |  | **D9** | 139 | 144 | 164 |
| **H10** | 141 | 143 | 163 |  | **D10** | 140 | 144 | 164 |
| **H11** | 140 | 145 | 164 |  | **D11** | 141 | 144 | 164 |
| **H12** | 140 | 143 | 164 |  | **D12** | 139 | 143 | 164 |
| **H13** | 138 | 143 | 163 |  | **D13** | 138 | 144 | 163 |
| **H14** | 142 | 146 | 164 |  | **D14** | 140 | 143 | 163 |
| **H15** | 139 | 143 | 164 |  | **D15** | 142 | 145 | 164 |
| **H16** | 141 | 143 | 164 |  | **D16** | 141 | 145 | 164 |
| **H17** | 142 | 143 | 164 |  | **D17** | 139 | 143 | 164 |
| **H18** | 141 | 144 | 164 |  | **D18** | 140 | 143 | 164 |
| **H19** | 141 | 144 | 164 |  | **D19** | 140 | 142 | 164 |
| **H20** | 138 | 143 | 164 |  | **D20** | 142 | 144 | 164 |
| **H21** | 140 | 146 | 164 |  | **D21** | 138 | 143 | 164 |
| **H22** | 140 | 143 | 164 |  | **D22** | 141 | 144 | 164 |
| **H23** | 139 | 144 | 164 |  | **D23** | 141 | 144 | 164 |
| **H24** | 139 | 145 | 164 |  | **D24** | 141 | 144 | 164 |
| **H25** | 141 | 143 | 164 |  | **D25** | 140 | 144 | 164 |
| **H26** | 140 | 143 | 165 |  | **D26** | 140 | 144 | 164 |
| **H27** | 140 | 143 | 164 |  | **D27** | 141 | 144 | 164 |
| **H28** | 136 | 144 | 164 |  | **D28** | 141 | 144 | 164 |
| **H29** | 138 | 144 | 164 |  | **D29** | 139 | 144 | 163 |
| **H30** | 139 | 143 | 164 |  | **D30** | 141 | 144 | 163 |
| **H31** | 140 | 144 | 163 |  | **D31** | 139 | 143 | 163 |
| **H32** | 141 | 145 | 165 |  | **D32** | 138 | 144 | 163 |
| **H33** | 139 | 143 | 164 |  | **D33** | 141 | 144 | 163 |
| **H34** | 141 | 145 | 164 |  | **D34** | 140 | 144 | 164 |
| **H35** | 141 | 144 | 164 |  | **D35** | 141 | 144 | 164 |
| **H36** | 140 | 144 | 164 |  | **D36** | 141 | 144 | 164 |
| **H37** | 139 | 143 | 164 |  | **D37** | 142 | 144 | 164 |
| **H38** | 141 | 144 | 164 |  | **D38** | 139 | 143 | 164 |
| **H39** | 139 | 144 | 164 |  | **D39** | 140 | 144 | 164 |
| **H40** | 141 | 144 | 163 |  | **D40** | 138 | 144 | 163 |
| **H41** | 140 | 144 | 163 |  | **D41** | 140 | 144 | 164 |
| **H42** | 141 | 143 | 164 |  | **D42** | 141 | 145 | 164 |
| **H43** | 139 | 143 | 164 |  | **D43** | 141 | 144 | 164 |
| **H44** | 141 | 144 | 164 |  | **D44** | 140 | 143 | 164 |
| **H45** | 139 | 144 | 164 |  | **D45** | 140 | 143 | 164 |
| **H46** | 141 | 143 | 164 |  | **D46** | 141 | 144 | 163 |
| **H47** | 142 | 145 | 164 |  | **D47** | 141 | 144 | 164 |
| **H48** | 140 | 144 | 164 |  | **D48** | 142 | 144 | 164 |
| **H49** | 139 | 143 | 163 |  | **D49** | 138 | 144 | 162 |
| **H50** | 139 | 144 | 164 |  | **D50** | 138 | 144 | 164 |

Table S3
